# Evolution of a key enzyme of aerobic metabolism reveals Proterozoic functional subunit duplication events and an ancient origin of animals

**DOI:** 10.1038/s41598-021-95094-4

**Published:** 2021-08-03

**Authors:** Bruno Santos Bezerra, Flavia Ariany Belato, Beatriz Mello, Federico Brown, Christopher J. Coates, Juliana de Moraes Leme, Ricardo I. F. Trindade, Elisa Maria Costa-Paiva

**Affiliations:** 1 Institute of Biosciences, Department of Zoology, University of Sao Paulo, São Paulo, Brazil; 2 Genetics Department, Biology Institute, Federal University of Rio de Janeiro, Rio de Janeiro, Brazil; 3 Department of Biosciences, Faculty of Science and Engineering, Swansea University, Swansea, UK; 4 Geoscience Institute, University of Sao Paulo, São Paulo, Brazil; 5 Institute of Astronomy, Geophysics and Atmospheric Sciences, University of Sao Paulo, São Paulo, Brazil

**Keywords:** Evolution, Molecular biology

## Abstract

The biological toolkits for aerobic respiration were critical for the rise and diversification of early animals. Aerobic life forms generate ATP through the oxidation of organic molecules in a process known as Krebs’ Cycle, where the enzyme isocitrate dehydrogenase (IDH) regulates the cycle's turnover rate. Evolutionary reconstructions and molecular dating of proteins related to oxidative metabolism, such as IDH, can therefore provide an estimate of when the diversification of major taxa occurred, and their coevolution with the oxidative state of oceans and atmosphere. To establish the evolutionary history and divergence time of NAD-dependent IDH, we examined transcriptomic data from 195 eukaryotes (mostly animals). We demonstrate that two duplication events occurred in the evolutionary history of NAD-IDH, one in the ancestor of eukaryotes approximately at 1967 Ma, and another at 1629 Ma, both in the Paleoproterozoic Era. Moreover, NAD-IDH regulatory subunits β and γ are exclusive to metazoans, arising in the Mesoproterozoic. Our results therefore support the concept of an ‘‘earlier-than-Tonian’’ diversification of eukaryotes and the pre-Cryogenian emergence of a metazoan IDH enzyme.

## Introduction

All living aerobic organisms need energy for their maintenance and growth—yielding ATP through the oxidation of organic molecules and using oxygen as the terminal electron acceptor^1^. The Tricarboxylic Acid (TCA) Cycle, also known as the Citric Acid Cycle or Krebs’ Cycle^2^ undertakes sequential oxidation. Considered a molecular furnace, the TCA does not simply play a role in ATP generation through catabolism of macromolecules, but it is inextricably linked to cellular functionality and homeostasis. The TCA (1) plays a unique role as an NAD and NADP coenzyme reducer for ATP production in the respiratory chain, (2) acts as a core metabolic integration point for catabolic processes of different macromolecules including carbohydrates, lipids, and proteins, and (3) produces intermediate components of anabolic processes, e.g., citrate that is used for lipogenesis^1,2^. The turnover of the TCA cycle and the accumulation of citrate is influenced by the enzyme isocitrate dehydrogenase (IDH), which catalyzes the often irreversible oxidative decarboxylation of isocitrate to alpha-ketoglutarate (and CO_2_), concurrent to coenzyme (NAD^+^, NADP) reduction^1^.

Across the evolution of life, IDH regulation became more complex and the proteins diversified in terms of subunit number, size, or coenzyme binding^3^. The TCA cycle in eukaryotes uses the hetero-oligomeric NAD-IDH that exclusively locates into the mitochondrial matrix, whereas prokaryotes use mostly the homodimeric enzyme NADP-IDH^4^. Eukaryotes also have homodimeric NADP-IDHs that do not function in the TCA cycle, but aid lipid metabolism and counter oxidative damage in the cytosol and mitochondria^5^. Overall, eukaryotic NAD-IDH and NADP-IDH show more complex allosteric regulation processes than the prokaryotic phosphorylation-mediated regulation of NADP-IDH^3,5–7^. Such differences in the eukaryotic and prokaryotic NADP-IDH allostery seem to have originated from two different mutation events in the NAD-dependent ancestor^8^. Eukaryotic NADP-IDHs regulate their activity by substrate induced conformational changes in the allosteric site in the presence of the substrate^5,7^. Conversely, prokaryotic NADP-IDHs loose activity when phosphate groups are added to specific serine residues in the presence of acetate, alpha-ketoglutarate, and NADPH^4^. Generally, the eukaryotic IDHs have more complex regulation processes than single-step phosphorylation/de-phosphorylation switches^3^.

Among lineages, we find distinct numbers of IDH subunits that have evolved to act as heteromeric protein assemblies. Fungal and green algal IDHs are formed by two different subunits: IDH1 associated with allosteric regulation of the enzyme, and IDH2 responsible for substrate catalysis^6,9^. IDH1 is also the name used for the cytosolic NADP-isocitrate dehydrogenase, whereas IDH2 is used for mitochondrial NADP-IDH (widely studied in humans). In the present study, we refer to IDH1 and IDH2 as the two subunits of the fungal NAD-dependent IDH. Metazoans, a lineage of opisthokonts (taxon composed by holozoans, including animals and fungi), present an exclusively octameric IDH with three different subunits: α, β and γ in a stoichiometric ratio 2:1:1^10^. The α subunit from mammals is structurally similar to the fungal IDH2 subunit, indicating that it has a catalytic function, whereas the β and γ subunits resemble the structure of the fungal IDH1 subunit, which serves as evidence of its regulatory function^11,12^.

Regarding animals, the need for oxygen is almost exclusively associated with aerobic respiration. Previous investigators have suggested a causal link between the diversification of animals and the rise of atmospheric dioxygen levels in the late Neoproterozoic (1.000–541 Ma)^13,14^. In fact, recent studies suggest that the earliest animals were likely small, soft-bodied, and collagen-poor—evolving under low oxygen levels that restricted their use of oxygen to high-priority physiological functions^15,16^, including basal cellular aerobic respiration. Moreover, much of the molecular toolkit required for animal development originated deep in eukaryote evolutionary history during low oxygen periods^17^.

The evolutionary history of animals could be traced by considering single and multicellular eukaryotes as well as animal phylogenies, and the divergence times among their major lineages. Even for the metazoan lineage, which is one of the best-studied eukaryotic lineages, a consensus does not exist regarding its basal relationships (e.g., ctenophore *vs* porifera controversy). Moreover, reconstructions of the last common ancestor for animals are contentious^18–21^, partly because early putative animal fossil records are problematic^22,23^. While molecular dating for crown Metazoa range between 1298 and 615 Ma, the earliest metazoan fossils date to the late Ediacaran ~ 580 Ma^24–26^. Fossils older than 600 Ma remain controversial and are limited to a few small sponges^27–29^. Regarding eukaryotes, molecular clock estimates for their last common ancestor span ~ 1 billion years^30–33^. Some differences can be attributed to the use of the fossil *Bangiomorpha pubescens*—a presumed bangiophyte red alga—as a calibration point. Time estimates obtained without employing *B. pubescens* as a calibration point are 200–300 Ma younger than those that do^34^. Recently, the development of refined molecular clock methodologies has helped to reduce the disparities among molecular dating and the fossil evidence used to estimate clade age minima^26,35^.

To elucidate the evolutionary processes that led to the diversification of major taxa during the geological history of the Earth, molecular estimates of divergence times are now used as a standard technique to infer chronograms^36^ and have improved dramatically in recent years for deep time studies^37–40^. The method consists of estimating the age of internal nodes based on the rates of nucleotide or amino acid substitutions among lineage sequences^36^. Understanding species phylogeny is a prerequisite for studying protein function and adaptations at the molecular level. In fact, the proteins of an organism share the same phylogenetic history, and changes in protein sequences can be used to date the origin of various physiological adaptations^41^. Therefore, molecular dating of specific genes can provide new insights into well-established topics, such as the diversification of eukaryotes and animals^26,42^. Although most deep time studies use hundreds of genes in order to estimate divergence times of species lineages^26,35^, molecular dating of particular proteins can recover the evolutionary history of these proteins against a background of the evolution of major taxa that are intrinsically linked to these proteins. Molecular dating of deep divergences may be challenging, mostly because of issues such as sequence saturation, which can affect analyses by biasing the estimated genetic distances^43–46^. However, estimated divergence times based on amino acid sequences that are more conserved compared to nucleotides sequences can alleviate the problem of saturation.

Because animals depend on the oxidation of organic molecules for cellular respiration, the evolution of proteins related to oxidative metabolism can allow us to trace their emergence time^47^. The crucial role of NAD-IDH for cellular respiration and Krebs’ Cycle^7^ makes its evolutionary history a compelling source of information to study the evolution of early animals and eukaryotes. Previous works have presented the phylogenetic relationships of the IDH protein family in Prokaryotes and Eukaryotes^8^, however the evolutionary history of NAD-IDHs in animals, as well as molecular dating estimates of protein divergence, are still unclear. Previously, authors described lineage-specific duplication events that gave rise to three IDH subunits (α, β and γ) in metazoans only^10^, and these events can be used to estimate the time of animal diversification^26^. We present the evolutionary history of eukaryotic NAD-IDHs and molecular dating estimates for the emergence of animal IDH and subsequent duplication events.

## Results

### Evolutionary history

**Figure 1:**
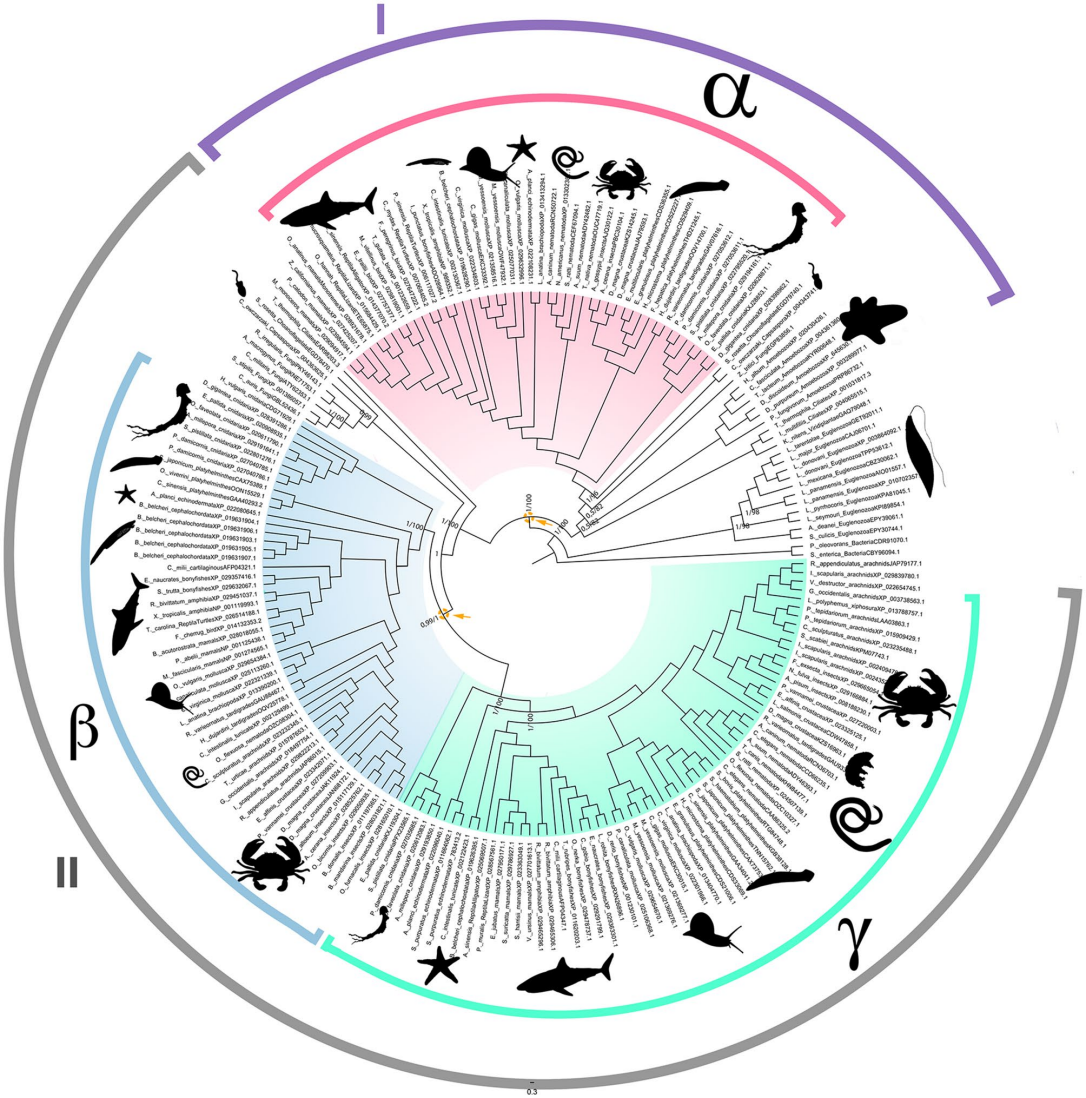
NAD-isocitrate dehydrogenase gene tree rooted with two bacterial sequences. Clade I is composed of eukaryotic catalytic subunits, and Clade II consists of eukaryotic regulatory subunits. The pink clade refers to metazoan catalytic subunit α, the blue clade refers to metazoan regulatory subunit β, and the green clade is the metazoan regulatory subunit γ. Arrows indicate gene duplication events that led to the evolution of NAD-IDH functional subunits. The number after the taxon name indicates the genetic identifier (GenBank) for each gene. Posterior probabilities and bootstrap support values are indicated for discussed nodes (PP/BP).

Our final empirical dataset comprised 193 IDH sequences from eukaryotes and two IDH sequences from bacteria, used here as the outgroup (Supplementary Table S1; Supplementary Figure S2). Following alignment and trimming, the dataset included 353 residues (alignment file available at 10.6084/m9.figshare.13158116). Bayesian inference and maximum likelihood analyses recovered the same topology for the main eukaryotic lineages with high support values, and also poorly resolved nodes that are often observed in gene genealogies^48^ (Fig. 1; IQ-tree tree file available at 10.6084/m9.figshare.13158101 and MrBayes tree file available at 10.6084/m9.figshare.13158110). Phylogenetic reconstruction performed with a mixture model, presented high concordance with Bayesian and maximum likelihood phylogenies based on regular substitution models (PhyloBayes tree file available at 10.6084/m9.figshare.14633157). In order to check if our heterogeneous taxonomic ensemble could bias the phylogenetic reconstructions, we tested the effect of taxonomic sampling by running a maximum likelihood analysis with only one representative for each taxon per subunit. The topology recovered was congruent with those obtained for our complete dataset.

Gene genealogy clearly evidenced multiple duplication events of eukaryotic NAD-IDH subunits that resulted in the three metazoan subunits, α, β and γ. IDHα was already present in the ancestor of metazoans, whereas IDHβ and IDHγ duplicated and diverged within the metazoan lineage (arrows, Fig. 1). IDHβ + IDHγ were sister groups to an unnamed IDH group—including Fungi, Capsaspora, and Choanoflagellate IDHs—forming a monophyletic opisthokonta clade. The opisthokonta IDHs showed a sister group relationship to another ciliate specific IDH that comprises the molecule in animals and the two subunits present in other opisthokonts (Fig. 1). This ciliate specific IDH likely originated from an earlier duplication event that pre-dated the opisthokonta ancestor.

The topology of the NAD-IDH gene tree corroborated the consensus of recent phylogenomic studies of Eukarya^49,50^. We found two highly supported clades (PP. = 1; BP = 100%): one comprised exclusively of IDH sequences from euglenozoans (Discoba) and the second contained all remaining sequences. The latter clade was formed by two sister-groups and displayed the first duplication event present in the eukaryotic NAD-IDH evolutionary history. These two well-supported clades were clade I containing catalytic subunits IDH2 and animal subunit α (Fig. 1; pink clade; PP = 1; BP = 100%) and clade II containing regulatory subunits IDH1 and animal subunits β + γ (Fig. 1; blue and green clades; PP > 0.9; BP > 95%).

The clade containing the catalytic NAD-IDH subunits (Fig. 1; pink clade) revealed the divergence between a clade comprising the ciliate IDH sequences (Fig. 1; PP. = 1; BP = 100%) and another composed of Amorphea (Opisthokonta + Amoebozoa) IDH sequences (Fig. 1; PP. > 0.5; BP > 80%). The latter is further divided into two sister-groups: amoebozoans (Fig. 1; PP. = 1; BP = 100%) and opisthokonts IDHs (Fig. 1; PP = 1; BP = 100%). The opisthokont clade is further subdivided into a single IDH2 fungal sequence and a holozoan clade (Fig. 1; PP = 1; BP = 100%). In the holozoan clade, we found two IDH sequences, one from a *Capsaspora* species and one from a choanoflagellate species, which was the sister clade to the metazoans (Fig. 1; PP = 1; BP = 100%). The genealogy of the catalytic subunit(s) recapitulated the recent phylogenetic relationships recovered for these organisms, suggesting a common evolutionary history of the gene and the events of lineage divergence in the opisthokonts.

Clade II—composed of the regulatory subunits—showed evidence for a second eukaryotic gene duplication event that gave rise to animal subunits β and γ (Fig. 1; blue and green clades; PP = 1.0; BP = 100%). Prior to this duplication event, another separation of two gene lineages occurred: one formed by a ciliate specific gene clade and another formed by IDH1 genes of choanoflagellates, capsasporans, and fungi (Fig. 1; PP > 0.9; BP > 80%). The second duplication event in the clade was associated exclusively with metazoan IDH sequences (Fig. 1; blue and green clades; PP > 0.9; BP > 95%). All metazoan lineages used in this study were represented in both the β subunit (Fig. 1; blue clade; PP > 0.9; BP > 95%) and γ subunit (Fig. 1; green clade; PP = 1.0; BP = 100%) clades.

### Molecular dating

The topology estimated by BEAST differed from the maximum-likelihood and Bayesian inferences. For instance, the euglenozoans NAD-IDH sequences formed a monophyletic clade within the catalytic subunit clade and only after the first eukaryotic duplication event. Moreover, a significant difference was observed in the position of choanoflagellate sequences in relation to animal subunits β and γ. In our former analysis (Fig. 1), IDH1 sequences of choanoflagellate, capsasporans, and fungi were the sister-group to animal subunits β and γ, prior to the second duplication event. In contrast, the BEAST analyses showed that the single choanoflagellate gene sequence appeared as a sister-group to animal subunit γ. With the exception of very deep divergences, node ages and credibility intervals estimated based on a mixture model in PhyloBayes generally matched the ones inferred by BEAST for the major clades (Supplementary Figure S3 and PhyloBayes timescale file available at 10.6084/m9.figshare.14633271). Because of this, we centered our results and discussion around BEAST’s timescale.

**Figure 2:**
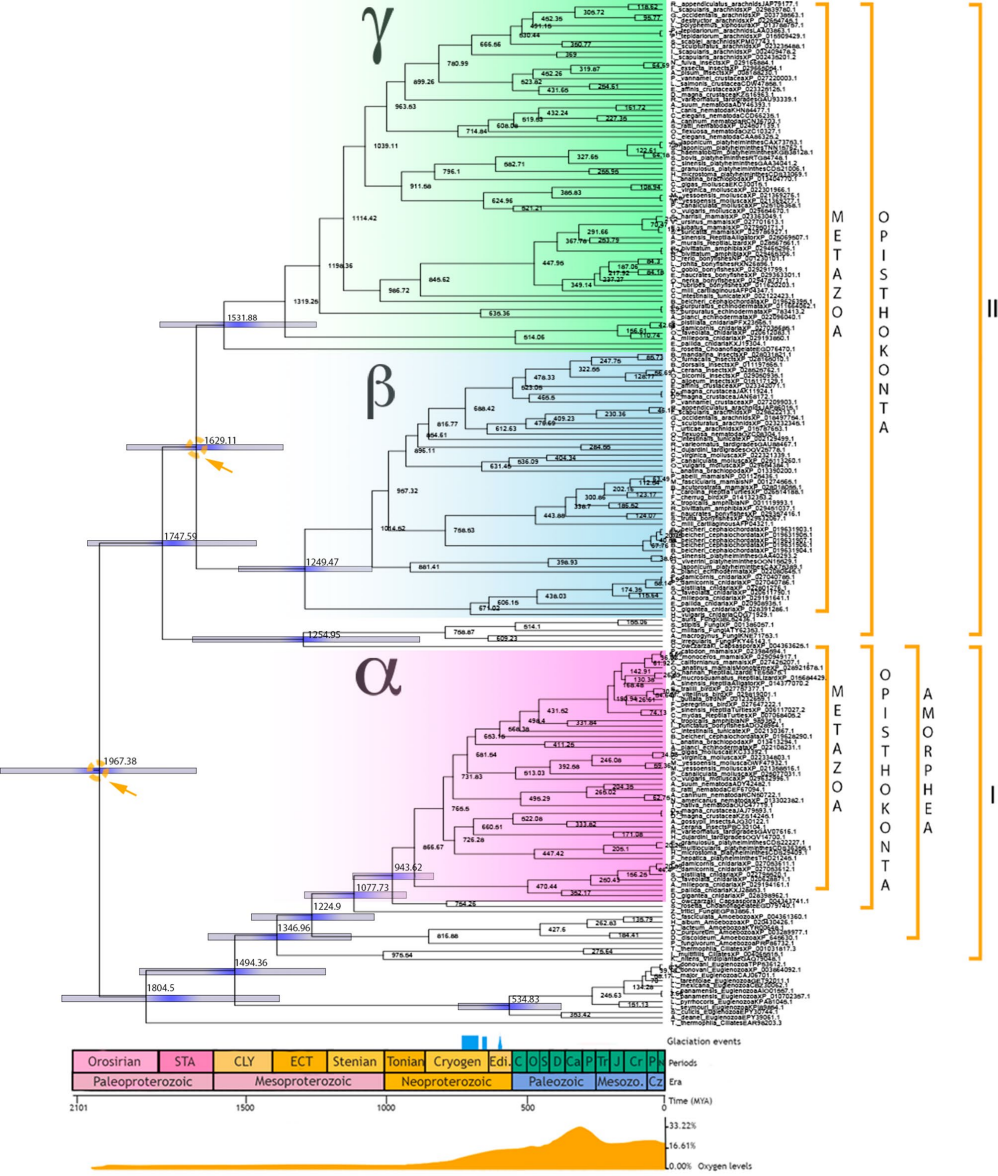
Timetree of NAD-isocitrate dehydrogenases. Clades I and II contain eukaryotic catalytic and regulatory subunits, respectively. The pink clade refers to metazoan catalytic subunit α, the blue clade refers to metazoan regulatory subunit β, and the green clade is the metazoan regulatory subunit γ. Node ages are plotted and node bars are displaying 95% HDP. STA, Statherian; CLY, Calymmian; ECT, Ectasian; Ediac, Ediacaran; C, Cambrian; O, Ordovician; S, Silurian; D, Devonian; Carb, Carboniferous, P, Permian; Tr, Triassic; J, Jurassic; K, Cretaceous; Pg, Paleogene; Ng, Neogene; Cz, Cenozoic; Ma, Million years ago. Glaciation events and atmospheric oxygen level estimates are indicated. The number after the taxon name indicates the genetic identifier (GenBank) for each gene.

The age estimated for the first duplication event of eukaryotic NAD-IDH giving rise to catalytic subunits (IDH2 and α) and regulatory subunits (IDH1, β and γ) was approximately 1967 Ma (1641–2329 Ma) in the Paleoproterozoic (Fig. 2). Regarding the catalytic subunit, the split between the euglenozoan subunit and other eukaryotes occurred at ~ 1494 Ma (1186–1838 Ma). The split of amoebozoan subunits and opisthokont catalytic subunit was dated at ~ 1224 Ma (1016–1447 Ma), whereas amoebozoan catalytic NAD-IDH was dated at ~ 816 Ma (538–1120 Ma) (Fig. 2). The estimated divergence date between choanoflagellates and capsasporan NAD-IDH and animal α NAD-IDH occurred at approximately 943 Ma (807–1089 Ma). Finally, the emergence of subunit α for animal NAD-IDH was dated at ~ 866 Ma (753–991 Ma), in the Neoproterozoic (Fig. 2).

The gene lineage that gave rise to eukaryotic regulatory subunits IDH1 and metazoan regulatory subunits β and γ diverged at approximately 1747 Ma (1431–2023 Ma), before the second eukaryotic gene duplication event. This divergence gave rise to a fungal and capsasporan IDH1 clade dated at ~ 1254 Ma (854–1651 Ma) and a metazoan IDHβ + IDHγ clade dated ~ 1629 Ma (1334–1884 Ma). The latter clade, composed of metazoan subunits β and γ, located the second gene duplication event. The origin of subunit β was estimated at ~ 1249 Ma (1020–1490 Ma), while the subunit γ was dated at ~ 1531 Ma (1221–1769 Ma). Although the subunit β is exclusive to animals, this analysis recovered a choanoflagellate IDH sequence as a sister group to animal subunit γ. Thus, the estimated age for the origin of animal subunit γ (without considering the choanoflagellate IDH sequence) was approximately 1319 Ma (1100–1558 Ma; Fig. 2).

## Discussion

**Figure 3:**
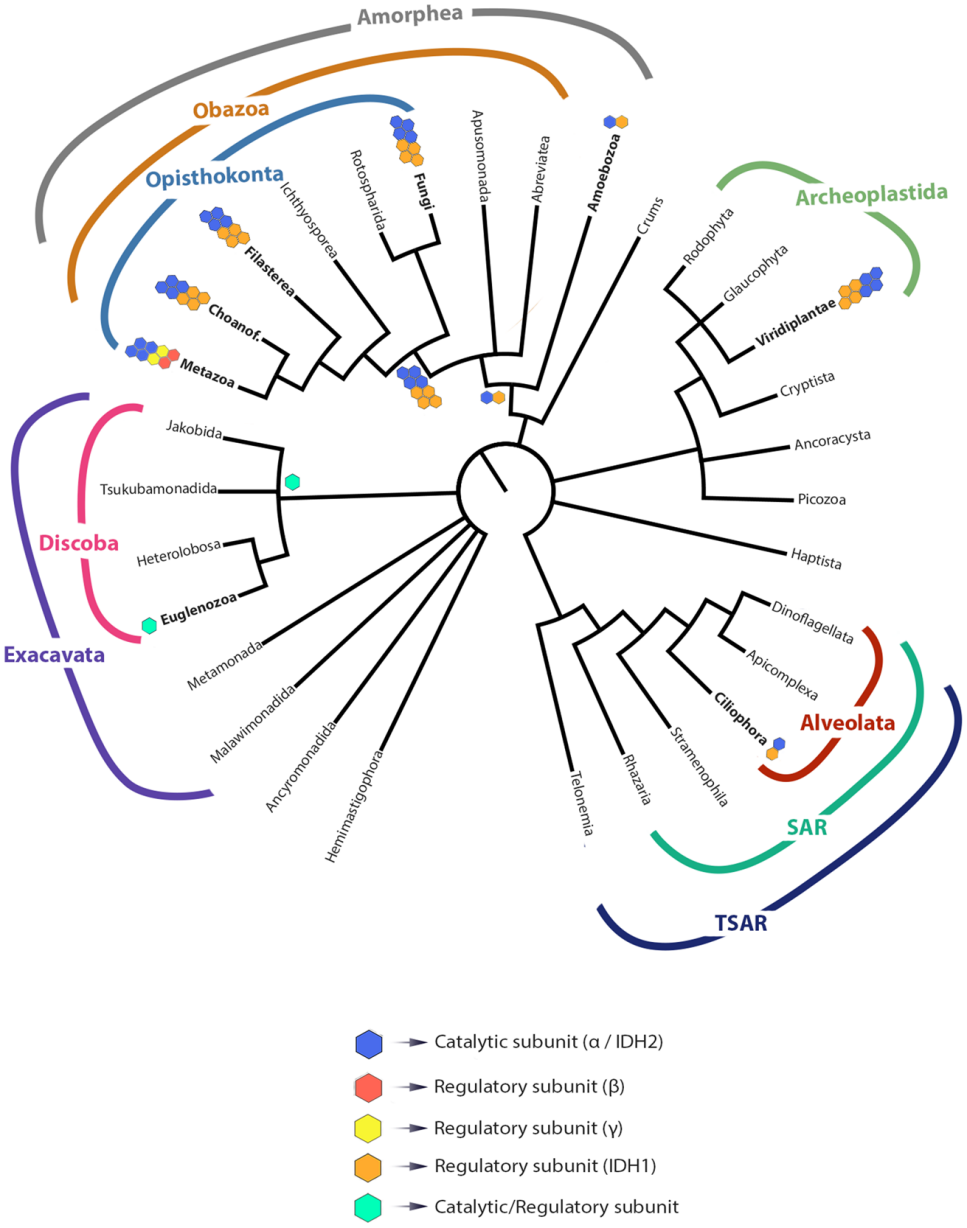
Hypothesized relationships among eukaryotes derived from recent phylogenomic studies^32,49,50^. Names in bold represent analyzed taxa. Colored hexagons represent NAD-IDH subunits.Blue hexagons represent catalytic subunit α or IDH1; red hexagons represent metazoan regulatory subunit β; yellow hexagons represent metazoan regulatory subunit γ; orange hexagons represent eukaryotic regulatory subunit IDH1; green hexagons represent the euglenozoan catalytic/regulatory subunit. The organization of hexagons represents the known octameric quaternary protein structure in fungi, filasterean, choanoflagellates, metazoans, and viridiplants. Oligomerisation remains unknown in ciliates.

We interrogated the emergence of metazoan NAD-IDH lineages in an attempt to resolve the relationships and evolutionary history of this protein among eukaryotic NAD-IDHs. Our results demonstrate that two gene duplication events occurred in the evolutionary history of NAD-IDH, one in the ancestor of eukaryotes approximately at 1967 Ma, and another at ~ 1629 Ma (both in the Paleoproterozoic Era). Additionally, we reveal that NAD-IDH regulatory subunits β and γ are exclusive to metazoans, and arose in the Mesoproterozoic. Previous studies have investigated the relationships between IDHs, focusing on NAD-IDH and NADP-IDH types^8,51^. Although the first members of NAD-IDHs were likely to be present in the eukaryotic ancestor, the quaternary structure of these enzymes remain unresolved. Early IDHs may have functioned as a homodimer as observed in living prokaryotes^4^, an octameric structure as observed in extant eukaryotes^3^, or as intermediate heterodimers. Previous evolutionary analyses indicated eubacterial IDHs first evolved from an NAD-dependent precursor about 3.5 Ga^8^. In the evolutionary history of this protein, our findings suggest that there was a tendency to increase regulatory complexity in eukaryotes, which concurs with other studies^3^ (Fig. 3). In bacteria, the regulation of IDH is achieved by simple phosphorylation events^4^, whereas eukaryotic IDHs contain at least two different subunits that show allosteric regulation by distinct factors^3^. Animals are the only eukaryotic group with three different subunits; more binding motifs that can trigger protein activity have been identified (e.g., ATP and Ca2^+^)^52,53^.

The ancestor of eukaryotic NAD-IDH arose ~ 1967 Ma (1641–2329 Ma) in the Paleoproterozoic. At that time, Earth was colonized mainly by microbial lifeforms, and representative species of only a few microbial clades are preserved in the fossil record. Moreover, the interpretation of these early eukaryote fossils is challenging because their key distinguishing characteristics, such as organelles and nuclei, do not preserve well^54^. Our findings for the emergence of eukaryotic NAD-IDH during the Paleoproterozoic is in agreement with contemporary molecular clock estimates for eukaryotes emergence^32,33^ and for the canonical view that stem and crown-group eukaryotes may have emerged early in the Proterozoic, keeping low diversity levels in restricted environments until a late Mesoproterozoic diversification^55–57^. Previous studies suggested that the last common eukaryotic ancestor lived between 1866 and 1679 Ma^32,58^, which is consistent with the earliest unambiguous microfossils interpreted as eukaryotic microfossils found in latest Paleoproterozoic rocks (ca. 1650 Ma)^59–61^. Additionally, the early development of the eukaryotic molecular toolkit originating from a stem–line of descent—a propagating series of pluripotent cellular entities—at approximately 2000 Ma continues to gather support in the literature^62^. Using molecular clock estimates of protein folds, the emergence of eukaryotes in the Paleoproterozoic is also corroborated^63,64^. Nevertheless, recent evidence suggest a late Mesoproterozoic origin of the eukaryotic crown group based mainly on eukaryotic sterols, which are presumed to have been present in the eukaryotic crown-ancestor and is absent from rock records until the early Neoproterozoic^34^. Although our results may support a deep Paleoproterozoic origin of eukaryotes with a late Mesoproterozoic origin of the crown group, they do not support a late emergence of aerobic respiration (ca. 800 Ma; as suggested by Porter et al.^34^).

The catalytic NAD-IDH subunit arose ca. 1967 Ma in the Paleoproterozoic as a result of the first gene duplication event in eukaryotes. This duplication event generated two NAD-IDHs, a subunit with catalytic function and another with regulatory function. According to our results, animal catalytic subunit α is closer to the fungal catalytic IDH2 sequences than to the other metazoan subunits β and γ, suggesting an orthologous relationship among animal α and fungal IDH2^11,12^. The emergence of the opisthokont NAD-IDH catalytic subunit during the Mesoproterozoic (ca. 1224 Ma) corroborates recent paleontological studies from rocks dated at 1700–800 Ma and suggests a higher eukaryotic diversity during this earlier interval than previously known^65–68^. The emergence of the amoebozoan NAD-IDH catalytic subunit was estimated to have occurred as recently as ~ 816 Ma in the Neoproterozoic, i.e., almost 700 Ma later than previous molecular dating estimates^32^. Amoebozoa is the eukaryotic supergroup sister to Obazoa, the group containing animals, fungi, and several microbial eukaryotic lineages^69,70^ (Fig. 3).

The emergence of the NAD-IDH regulatory subunit was dated at ~ 1747 Ma, giving rise to two distinct gene lineages: a fungi + capsasporan NAD-IDH and a metazoan NAD-IDH β + γ. The emergence of the fungi/capsasporan NAD-IDH regulatory subunit occurred in the Mesoproterozoic and was dated at ~ 1254 Ma, corroborating previous estimates for the last common ancestor of extant Opisthokonta from 1389 to 1240 Ma^32^. The regulatory subunit IDH1 in fungi is closely related to subunits β and γ in animals, indicating that a second duplication event occurred. This result corroborates previously identified physiochemical properties between fungi and mammalian NAD-IDH sequences, such as, comparable molecular weights of subunits and active site architecture^12,53^.

The NAD-IDH regulatory subunit originated from a gene duplication event in the Paleoproterozoic (ca. 1967 Ma). However, the emergence of the lineage that gave rise to metazoan subunits β + γ occurred later in the Mesoproterozoic (ca. 1629 Ma). Dates of emergence of animal-specific NAD-IDH subunit β (~ 1249 Ma) and NAD-IDH subunit γ (~ 1532 Ma) provide support for an earlier emergence of metazoans compared to previous estimates that date their origin to ~ 800 Ma^71,72^. If animal divergence began in the Mesoproterozoic or even later in the Neoproterozoic, as suggested by recent molecular estimates^26,35,73^, hypoxic conditions should have prevailed in most environmental settings for more than half of animal evolutionary history^74^. An alternative scenario could be the existence of oxygenic niches present at the microscale since the rise of oxidative photosynthesis about 3.0 Ga^74,75^. In spite of the strong debate surrounding those conditions^76–79^, the fact is that hypoxic niches may have prevailed for most of Earth’s history. Thus, a long-lasting hypoxic period of animal evolution may have resulted in particular adaptations to meet the metabolic demands of oxygen, which could explain an evolutionary trend towards an increase of complexity in the NAD-IDH molecule. Beyond IDHs, tracing the presence/absence and evolutionary history of oxygen reductases (*e.g*., complex IV and cytochrome bd) would offer further insight into the prototypical pathways of aerobic metabolism in early metazoans when oxygen availability was a limiting factor.

Overall, our results support the hypothesis of an ‘‘earlier-than-Tonian’’ diversification of eukaryotes, as well as a pre-Cryogenian emergence of metazoans under low-oxygen conditions. This implies there has been an evolutionary trend towards increasing complexity in the regulatory subunit of animal NAD-IDH, which includes a complex of three subunits (one catalytic and two regulatory subunits). Our study also highlights the importance of molecular dating estimates of protein families for enhancing our understanding of evolution in deep time.

## Methods

### Sequence retrieval

The dataset consisted of 195 protein sequences obtained from the National Centre for Biotechnological Information (NCBI) database (Supplementary Table S1; Supplementary Figure S2). Considering that the accuracy of a phylogeny protocol is strongly dependent on the taxonomic sampling, number of sequences and the sequence length, we bolstered our dataset by searching for as many sequences from all metazoan phyla available at NCBI. Sequences functionally annotated as “isocitrate dehydrogenase” in the protein database were retrieved with the following criteria used to filter the appropriate sequences: 1) for taxa with more than 15 sequences available in NCBI, those sequences that were denoted as “Putative”, “Hypothetical”, “Low-quality”, and “partial” were removed; 2) sequences with fewer than 350 amino acid residues were also removed. To confirm the protein domain identity of the sequences, a local search using HMMER 3.3 was performed using the Pfam database with an e-value cutoff of 1e-5^80^. Sequences remaining after those validation steps were incorporated into the final dataset for analysis.

### Alignments and phylogenetic reconstructions

In order to infer homology between the sequences, we aligned the dataset using MAFFT software^81^ with the accurate algorithm “G-INS-i”. To obtain a biologically correct alignment, we performed a visual inspection and a manual curation to remove spuriously aligned sequences, based on similarity to the protein alignment as a whole. In order to eliminate poorly aligned regions, the alignment was trimmed using the software trimAl^82^ with a 75% gap threshold. To eliminate redundancy from the dataset, sequences with 100% similarity to each other after trimming were removed. The resulted alignment was used for downstream analysis.

Phylogenetic reconstructions rely on the assumption of empirical models of substitution and are therefore dependent on the correct choice of those models. Thus, the best-fit model of protein evolution for the dataset was selected using ModelFinder, implemented in the IQ-TREE software^83^, which uses Akaike and Bayesian Information Criteria methods. IQ-TREE was also used to perform a maximum likelihood inference^84^. The branch supports and the robustness of the analyses were obtained by using an ultrafast bootstrap approximation with 1000 replicates^85^. Moreover, we also performed Bayesian inference with MrBayes 3.2.1^86^ using two independent runs, each with four Metropolis-coupled chains for 10^7^ generations, sampling from the posterior distribution every 500 generations. To confirm whether chains achieved stationary and to determine an appropriate burn-in, we evaluated trace plots of all MrBayes parameter outputs in Tracer v1.6^87^. The first 25% of samples were discarded as burn-in and a majority rule consensus tree was generated. The software FigTree 1.4.3^88^ was used to summarize and root the resulting phylogenetic trees using two sequences of isocitrate dehydrogenase from bacteria as an outgroup. Because of the evolutionary depth of our dataset, the phylogenetic reconstruction was also performed with a mixture model, which alleviates the issues related to saturation and long branch attraction by accommodating site-specific features of protein evolution^89,90^. This was done in PhyloBayes^89^ by using the CAT-GTR model with a gamma distribution (Γ4) of site-rate heterogeneity and multiple chains to check for convergence.

Divergence time estimates were obtained using the software BEAST 2.4.7^91^ with the uncorrelated lognormal relaxed clock^92^ using the LG substitution model^93^, and a birth–death tree prior with default settings. To calibrate divergence times, only speciation nodes were considered because calibration information derived from fossil data provides information regarding the split times between biological lineages (*i.e.,* speciation events). Therefore, divergences classified as speciation nodes that reflected robust biological clades and were free of duplication events were chosen for calibration. These were the crown nodes of Pancrustacea and Gnathostomata. For both taxa, the aforementioned requirements were met three times (*i.e.,* speciation nodes that included only Pancrustacea or Gnathostomata NAD-IDH sequences were recovered three times in the estimated phylogeny). Because of that, six speciation nodes were calibrated with uniform distributions with lower and upper boundaries based on estimates from Benton et al.^94^ and dos Reis et al.^26^. We used time ranges of 514–531.22 Ma and 420.7 to 468.4 Ma to calibrate the tMRCA (time to the Most Recent Common Ancestor) of pancrustaceans and gnathostomes, respectively. Our calibration points were determined using coherent criteria according to Parham et al.^95^. It is worth mentioning that calibrated nodes were constrained to monophyletic, while other phylogenetic relationships were estimated in BEAST. MCMC (Markov Chain Monte Carlo) models were run for 200 million generations with a sampling frequency of 10,000 and a discarded burn-in period of 10% (20 million generations). To access convergence of chains, two independent MCMC runs were performed. In both runs, effective sample size values were higher than 200 after discarding the burn-in period. We also estimated divergence times in PhyloBayes by using the UGAM relaxed clock model and the phylogeny estimated previously in PhyloBayes by running multiple chains to check for convergence. To reduce computational burden, the CAT-Poisson mixture model was used with a gamma distribution (Γ4) of site-rate heterogeneity. As in BEAST, calibrations were provided as uniform distributions. Because PhyloBayes requires a root calibration, we used a loose gamma distribution (mean = 1520, SD = 240) to calibrate the divergence between Euglenozoa and the remaining eukaryotes, which was based on estimated times retrieved from the TimeTree database^96^.

## Supplementary information

Supplementary Informations

## Data Availability

The datasets generated and analyzed during the current study are available in the FigShare repository. IQ-tree tree file available at 10.6084/m9.figshare.13158101, MrBayes tree file available at 10.6084/m9.figshare.13158110 and alignment file available at 10.6084/m9.figshare.13158116. PhyloBayes tree file available at 10.6084/m9.figshare.14633157 and PhyloBayes timescale file available at 10.6084/m9.figshare.14633271.
