# Supplementary material for: Evolution of a key enzyme of aerobic metabolism reveals Proterozoic functional subunit duplication events and an ancient origin of animals
Source: Sci Rep. 2021 Aug 3;11:15744. doi: 10.1038/s41598-021-95094-4 (PMC8333347; doi:10.1038/s41598-021-95094-4)

**Evolution of a key enzyme of aerobic metabolism reveals Proterozoic functional subunit duplication events and an ancient origin of animals**

Bruno Santos Bezerra[1], Flavia Ariany Belato[1], Beatriz Mello[2], Federico Brown[1], Christopher J. Coates[3], Juliana de Moraes Leme[4], Ricardo I. F. Trindade[5] & Elisa Maria Costa-Paiva[1,5*]

1. Institute of Biosciences, Department of Zoology, University of Sao Paulo, Brazil

2. Biology Institute, Genetics Department, Federal University of Rio de Janeiro, Brazil

3. Department of Biosciences, College of Science, Swansea University, United Kingdom

4. Geoscience Institute, University of Sao Paulo, Brazil

5. Institute of Astronomy, Geophysics and Atmospheric Sciences, University of Sao Paulo, Brazil

Corresponding author: elisam.costapaiva@gmail.com

**Supplementary Table S1** – List of all taxa analyzed in this study, including sequence name, species name, taxonomic group and GenBank accession number.

| Sequence Name | Species | Taxonomic Group | Accession number |
|---|---|---|---|
| A._planci_echinodermataXP_022080645.1 | *Acanthaster planci* | Echinodermata | XP_022080645.1 |
| A._planci_echinodermataXP_022096040.1 | *Acanthaster planci* | Echinodermata | XP_022096040.1 |
| A._planci_echinodermataXP_022108231.1 | *Acanthaster planci* | Echinodermata | XP_022108231.1 |
| A._millepora_cnidariaXP_029191641.1 | *Acropora millepora* | Cnidaria | XP_029191641.1 |
| A._millepora_cnidariaXP_029193850.1 | *Acropora millepora* | Cnidaria | XP_029193850.1 |
| A._millepora_cnidariaXP_029194161.1 | *Acropora millepora* | Cnidaria | XP_029194161.1 |
| A._pisum_insectsXP_008188230.1 | *Acyrthosiphon pisum* | Arthropoda | XP_008188230.1 |
| A._sinensis_ReptilaAlligatorXP_014377070.2 | *Alligator sinensis* | Vertebrata | XP_014377070.2 |
| A._sinensis_ReptilaAlligatorXP_025069507.1 | *Alligator sinensis* | Vertebrata | XP_025069507.1 |
| A._macrogynus_FungiKNE71753.1 | *Allomyces macrogynus* | Fungi | KNE71753.1 |
| A._caninum_nematodaRCN36703.1 | *Ancylostoma caninum* | Nematoda | RCN36703.1 |
| A._caninum_nematodaRCN50722.1 | *Ancylostoma caninum* | Nematoda | RCN50722.1 |
| A._deanei_EuglenozoaEPY39061.1 | *Angomonas deanei* | Euglenozoa | EPY39061.1 |
| A._gossypii_insectsAJQ30122.1 | *Aphis gossypii* | Arthropoda | AJQ30122.1 |
| A._cerana_insectsXP_028525762.1 | *Apis cerana* | Arthropoda | XP_028525762.1 |
| A._cerana_insectsPBC30104.1 | *Apis cerana cerana* | Arthropoda | PBC30104.1 |
| A._suum_nematodaADY42482.1 | *Ascaris suum* | Nematoda | ADY42482.1 |
| A._suum_nematodaADY46393.1 | *Ascaris suum* | Nematoda | ADY46393.1 |
| B._dorsalis_insectsXP_011197565.1 | *Bactrocera dorsalis* | Arthropoda | XP_011197565.1 |
| B._scammoni_mamalsXP_028018055.1 | *Balaenoptera acutorostrata scammoni* | Vertebrata | XP_028018055.1 |
| B._mandarina_insectsXP_028031821.1 | *Bombyx mandarina* | Arthropoda | XP_028031821.1 |
| B._belcheri_cephalochordataXP_019626395.1 | *Branchiostoma belcheri* | Cephalochordata | XP_019626395.1 |
| B._belcheri_cephalochordataXP_019628290.1 | *Branchiostoma belcheri* | Cephalochordata | XP_019628290.1 |
| B._belcheri_cephalochordataXP_019631903.1 | *Branchiostoma belcheri* | Cephalochordata | XP_019631903.1 |
| B._belcheri_cephalochordataXP_019631904.1 | *Branchiostoma belcheri* | Cephalochordata | XP_019631904.1 |
| B._belcheri_cephalochordataXP_019631905.1 | *Branchiostoma belcheri* | Cephalochordata | XP_019631905.1 |

| | | | |
|---|---|---|---|
| B._belcheri_cephalochordataXP_019631906.1 | *Branchiostoma belcheri* | Cephalochordata | XP_019631906.1 |
| B._belcheri_cephalochordataXP_019631907.1 | *Branchiostoma belcheri* | Cephalochordata | XP_019631907.1 |
| C._elegans_nematodaCAA86325.2 | *Caenorhabditis elegans* | Nematoda | CAA86325.2 |
| C._elegans_nematodaCCD66235.1 | *Caenorhabditis elegans* | Nematoda | CCD66235.1 |
| C._milii_cartilaginousAFP04321.1 | *Callorhinchus milii* | Vertebrata | AFP04321.1 |
| C._milii_cartilaginousAFP04347.1 | *Callorhinchus milii* | Vertebrata | AFP04347.1 |
| C._auris_FungiGBL52436.1 | *Candida auris* | Fungi | GBL52436.1 |
| C._owczarzaki_CapsasporaXP_004343741.1 | *Capsaspora owczarzaki* | Filasterea | XP_004343741.1 |
| C._owczarzaki_CapsasporaXP_004363625.1 | *Capsaspora owczarzaki* | Filasterea | XP_004363625.1 |
| C._fasciculata_AmoebozoaXP_004361360.1 | *Cavenderia fasciculata* | Amoebozoa | XP_004361360.1 |
| C._sculpturatus_arachnidsXP_023232345.1 | *Centruroides sculpturatus* | Arthropoda | XP_023232345.1 |
| C._sculpturatus_arachnidsXP_023235488.1 | *Centruroides sculpturatus* | Arthropoda | XP_023235488.1 |
| C._mydas_ReptilaTurtlesXP_007068405.2 | *Chelonia mydas* | Vertebrata | XP_007068405.2 |
| C._intestinalis_tunicateXP_002122423.1 | *Ciona intestinalis* | Urochordata | XP_002122423.1 |
| C._intestinalis_tunicateXP_002129499.1 | *Ciona intestinalis* | Urochordata | XP_002129499.1 |
| C._intestinalis_tunicateXP_002130367.1 | *Ciona intestinalis* | Urochordata | XP_002130367.1 |
| C._sinensis_platyhelminthesGAA34041.2 | *Clonorchis sinensis* | Platyhelminthes | GAA34041.2 |
| C._sinensis_platyhelminthesGAA40293.2 | *Clonorchis sinensis* | Platyhelminthes | GAA40293.2 |
| C._militaris_FungiATY62353.1 | *Cordyceps militaris* | Fungi | ATY62353.1 |
| C._gobio_bonyfishesXP_029291799.1 | *Cottoperca gobio* | Vertebrata | XP_029291799.1 |
| C._gigas_molluscaEKC30015.1 | *Crassostrea gigas* | Mollusca | EKC30015.1 |
| C._gigas_molluscaEKC33392.1 | *Crassostrea gigas* | Mollusca | EKC33392.1 |
| C._virginica_molluscaXP_022301966.1 | *Crassostrea virginica* | Mollusca | XP_022301966.1 |
| C._virginica_molluscaXP_022321339.1 | *Crassostrea virginica* | Mollusca | XP_022321339.1 |
| C._virginica_molluscaXP_022334803.1 | *Crassostrea virginica* | Mollusca | XP_022334803.1 |
| D._rerio_bonyfishesNP_001230101.1 | *Danio rerio* | Vertebrata | NP_001230101.1 |
| D._magna_crustaceaJAJ79593.1 | *Daphnia magna* | Arthropoda | JAJ79593.1 |
| D._magna_crustaceaJAK11924.1 | *Daphnia magna* | Arthropoda | JAK11924.1 |
| D._magna_crustaceaJAN68172.1 | *Daphnia magna* | Arthropoda | JAN68172.1 |
| D._magna_crustaceaKZS14245.1 | *Daphnia magna* | Arthropoda | KZS14245.1 |
| D._magna_crustaceaKZS16963.1 | *Daphnia magna* | Arthropoda | KZS16963.1 |

| | | | |
|---|---|---|---|
| D._gigantea_cnidariaXP_028391286.1 | *Dendronephthya gigantea* | Cnidaria | XP_028391286.1 |
| D._gigantea_cnidariaXP_028398962.1 | *Dendronephthya gigantea* | Cnidaria | XP_028398962.1 |
| D._alloeum_insectsXP_015117129.1 | *Diachasma alloeum* | Arthropoda | XP_015117129.1 |
| D._discoideum_AmoebozoaXP_645630.1 | *Dictyostelium discoideum* | Amoebozoa | XP_645630.1 |
| D._purpureum_AmoebozoaXP_003289977.1 | *Dictyostelium purpureum* | Amoebozoa | XP_003289977.1 |
| E._naucrates_bonyfishesXP_029357416.1 | *Echeneis naucrates* | Vertebrata | XP_029357416.1 |
| E._naucrates_bonyfishesXP_029363301.1 | *Echeneis naucrates* | Vertebrata | XP_029363301.1 |
| E._granulosus_platyhelminthesCDS21006.1 | *Echinococcus granulosus* | Platyhelminthes | CDS21006.1 |
| E._granulosus_platyhelminthesCDS22227.1 | *Echinococcus granulosus* | Platyhelminthes | CDS22227.1 |
| E._multilocularis_platyhelminthesCDS36355.1 | *Echinococcus multilocularis* | Platyhelminthes | CDS36355.1 |
| E._traillii_birdXP_027757377.1 | *Empidonax traillii* | Vertebrata | XP_027757377.1 |
| E._jubatus_mamalsXP_027950171.1 | *Eumetopias jubatus* | Vertebrata | XP_027950171.1 |
| E._affinis_crustaceaXP_023325125.1 | *Eurytemora affinis* | Arthropoda | XP_023325125.1 |
| E._affinis_crustaceaXP_023342071.1 | *Eurytemora affinis* | Arthropoda | XP_023342071.1 |
| E._pallida_cnidariaKXJ19304.1 | *Exaiptasia pallida* | Cnidaria | KXJ19304.1 |
| E._pallida_cnidariaKXJ28853.1 | *Exaiptasia pallida* | Cnidaria | KXJ28853.1 |
| E._pallida_cnidariaXP_020908935.1 | *Exaiptasia pallida* | Cnidaria | XP_020908935.1 |
| F._cherrug_birdXP_014132353.2 | *Falco cherrug* | Vertebrata | XP_014132353.2 |
| F._peregrinus_birdXP_027647222.1 | *Falco peregrinus* | Vertebrata | XP_027647222.1 |
| F._hepatica_platyhelminthesTHD21245.1 | *Fasciola hepatica* | Platyhelminthes | THD21245.1 |
| F._exsecta_insectsXP_029665054.1 | *Formica exsecta* | Arthropoda | XP_029665054.1 |
| G._occidentalis_arachnidsXP_003738563.1 | *Galendromus occidentalis* | Arthropoda | XP_003738563.1 |
| G._occidentalis_arachnidsXP_018497754.1 | *Galendromus occidentalis* | Arthropoda | XP_018497754.1 |
| H._album_AmoebozoaXP_020430426.1 | *Heterostelium album* | Amoebozoa | XP_020430426.1 |
| H._vulgaris_cnidariaCDG71929.1 | *Hydra vulgaris* | Cnidaria | CDG71929.1 |
| H._microstoma_platyhelminthesCDS29409.1 | *Hymenolepis microstoma* | Platyhelminthes | CDS29409.1 |
| H._microstoma_platyhelminthesCDS33069.1 | *Hymenolepis microstoma* | Platyhelminthes | CDS33069.1 |
| H._dujardini_tardigradesOQV14700.1 | *Hypsibius dujardini* | Tardigrada | OQV14700.1 |
| H._dujardini_tardigradesOQV25778.1 | *Hypsibius dujardini* | Tardigrada | OQV25778.1 |

| | | | |
|---|---|---|---|
| I._multifiliis_CiliatesXP_004065515.1 | *Ichthyophthirius multifiliis* | Ciliophora | XP_004065515.1 |
| I._punctatus_bonyfishesADO28964.1 | *Ictalurus punctatus* | Vertebrata | ADO28964.1 |
| I._scapularis_arachnidsXP_002409478.2 | *Ixodes scapularis* | Arthropoda | XP_002409478.2 |
| I._scapularis_arachnidsXP_002435201.2 | *Ixodes scapularis* | Arthropoda | XP_002435201.2 |
| I._scapularis_arachnidsXP_029822213.1 | *Ixodes scapularis* | Arthropoda | XP_029822213.1 |
| I._scapularis_arachnidsXP_029839780.1 | *Ixodes scapularis* | Arthropoda | XP_029839780.1 |
| K._nitens_ViridiplantaeGAQ79048.1 | *Klebsormidium nitens* | Viridiplantae | GAQ79048.1 |
| L._rohita_bonyfishesRXN26896.1 | *Labeo rohita* | Vertebrata | RXN26896.1 |
| L._donovani_EuglenozoaTPP53612.1 | *Leishmania donovani* | Euglenozoa | TPP53612.1 |
| L._donovani_EuglenozoaXP_003864092.1 | *Leishmania donovani* | Euglenozoa | XP_003864092.1 |
| L._major_EuglenozoaCAJ06701.1 | *Leishmania* major strain Friedlin | Euglenozoa | CAJ06701.1 |
| L._mexicana_EuglenozoaCBZ30062.1 | *Leishmania mexicana* | Euglenozoa | CBZ30062.1 |
| L._panamensis_EuglenozoaAIO01557.1 | *Leishmania panamensis* | Euglenozoa | AIO01557.1 |
| L._panamensis_EuglenozoaXP_010702357.1 | *Leishmania panamensis* | Euglenozoa | XP_010702357.1 |
| L._tarentolae_EuglenozoaGET92011.1 | *Leishmania tarentolae* | Euglenozoa | GET92011.1 |
| L._salmonis_crustaceaCDW47858.1 | *Lepeophtheirus salmonis* | Arthropoda | CDW47858.1 |
| L._pyrrhocoris_EuglenozoaKPA81045.1 | *Leptomonas pyrrhocoris* | Euglenozoa | KPA81045.1 |
| L._seymouri_EuglenozoaKPI89854.1 | *Leptomonas seymouri* | Euglenozoa | KPI89854.1 |
| L._polyphemus_xiphosuraXP_013788757.1 | *Limulus polyphemus* | Arthropoda | XP_013788757.1 |
| L._anatina_brachiopodaXP_013390200.1 | *Lingula anatina* | Brachiopoda | XP_013390200.1 |
| L._anatina_brachiopodaXP_013404770.1 | *Lingula anatina* | Brachiopoda | XP_013404770.1 |
| L._anatina_brachiopodaXP_013413294.1 | *Lingula anatina* | Brachiopoda | XP_013413294.1 |
| M._fascicularis_mamalsNP_001274565.1 | *Macaca fascicularis* | Vertebrata | NP_001274565.1 |
| M._vitellinus_birdXP_029819001.1 | *Manacus vitellinus* | Vertebrata | XP_029819001.1 |
| M._yessoensis_molluscaOWF47932.1 | *Mizuhopecten yessoensis* | Mollusca | OWF47932.1 |
| M._yessoensis_molluscaXP_021358516.1 | *Mizuhopecten yessoensis* | Mollusca | XP_021358516.1 |
| M._yessoensis_molluscaXP_021369276.1 | *Mizuhopecten yessoensis* | Mollusca | XP_021369276.1 |
| M._yessoensis_molluscaXP_021369277.1 | *Mizuhopecten yessoensis* | Mollusca | XP_021369277.1 |
| M._monoceros_mamalsXP_029094917.1 | *Monodon monoceros* | Vertebrata | XP_029094917.1 |
| N._americanus_nematodaXP_013302382.1 | *Necator americanus* | Nematoda | XP_013302382.1 |

| | | | |
|---|---|---|---|
| N._fulva_insectsXP_029166884.1 | *Nylanderia fulva* | Arthropoda | XP_029166884.1 |
| O._vulgaris_molluscaXP_029632996.1 | *Octopus vulgaris* | Mollusca | XP_029632996.1 |
| O._vulgaris_molluscaXP_029654384.1 | *Octopus vulgaris* | Mollusca | XP_029654384.1 |
| O._vulgaris_molluscaXP_029654670.1 | *Octopus vulgaris* | Mollusca | XP_029654670.1 |
| O._flexuosa_nematodaOZC08304.1 | *Onchocerca flexuosa* | Nematoda | OZC08304.1 |
| O._flexuosa_nematodaOZC10327.1 | *Onchocerca flexuosa* | Nematoda | OZC10327.1 |
| O._nerka_bonyfishesXP_029478737.1 | *Oncorhynchus nerka* | Vertebrata | XP_029478737.1 |
| O._hannah_ReptilaLizardETE65875.1 | *Ophiophagus hannah* | Vertebrata | ETE65875.1 |
| O._viverrini_platyhelminthesOON15529.1 | *Opisthorchis viverrini* | Platyhelminthes | OON15529.1 |
| O._faveolata_cnidariaXP_020611790.1 | *Orbicella faveolata* | Cnidaria | XP_020611790.1 |
| O._faveolata_cnidariaXP_020612083.1 | *Orbicella faveolata* | Cnidaria | XP_020612083.1 |
| O._faveolata_cnidariaXP_020628871.1 | *Orbicella faveolata* | Cnidaria | XP_020628871.1 |
| O._anatinus_mamalsMonotremeXP_028921678.1 | *Ornithorhynchus anatinus* | Vertebrata | XP_028921678.1 |
| O._bicornis_insectsXP_029050935.1 | *Osmia bicornis bicornis* | Arthropoda | XP_029050935.1 |
| O._furnacalis_insectsXP_028165010.1 | *Ostrinia furnacalis* | Arthropoda | XP_028165010.1 |
| P._tepidariorum_arachnidsLAA03863.1 | *Parasteatoda tepidariorum* | Arthropoda | LAA03863.1 |
| P._tepidariorum_arachnidsXP_015909429.1 | *Parasteatoda tepidariorum* | Arthropoda | XP_015909429.1 |
| P._sinensis_ReptilaTurtlesXP_006117027.2 | *Pelodiscus sinensis* | Vertebrata | XP_006117027.2 |
| P._vannamei_crustaceaXP_027209903.1 | *Penaeus vannamei* | Arthropoda | XP_027209903.1 |
| P._vannamei_crustaceaXP_027220003.1 | *Penaeus vannamei* | Arthropoda | XP_027220003.1 |
| P._catodon_mamalsXP_023984594.1 | *Physeter catodon* | Vertebrata | XP_023984594.1 |
| P._fungivorum_AmoebozoaPRP86732.1 | *Planoprotostelium fungivorum* | Amoebozoa | PRP86732.1 |
| P._damicornis_cnidariaXP_027035685.1 | *Pocillopora damicornis* | Cnidaria | XP_027035685.1 |
| P._damicornis_cnidariaXP_027040785.1 | *Pocillopora damicornis* | Cnidaria | XP_027040785.1 |
| P._damicornis_cnidariaXP_027040786.1 | *Pocillopora damicornis* | Cnidaria | XP_027040786.1 |
| P._damicornis_cnidariaXP_027053611.1 | *Pocillopora damicornis* | Cnidaria | XP_027053611.1 |
| P._damicornis_cnidariaXP_027053612.1 | *Pocillopora damicornis* | Cnidaria | XP_027053612.1 |
| P._muralis_ReptilaLizardXP_028567561.1 | *Podarcis muralis* | Vertebrata | XP_028567561.1 |
| P._canaliculata_molluscaXP_025077031.1 | *Pomacea canaliculata* | Mollusca | XP_025077031.1 |
| P._canaliculata_molluscaXP_025106368.1 | *Pomacea canaliculata* | Mollusca | XP_025106368.1 |

| | | | |
|---|---|---|---|
| P._canaliculata_molluscaXP_025113260.1 | *Pomacea canaliculata* | Mollusca | XP_025113260.1 |
| P._abelii_mamalsNP_001125436.1 | *Pongo abelii* | Vertebrata | NP_001125436.1 |
| P._mucrosquamatus_ReptilaLizardXP_015684429.1 | *Protobothrops mucrosquamatus* | Vertebrata | XP_015684429.1 |
| P._oleovorans_BacteriaCDR91070.1 | *Pseudomonas oleovorans* | Bacteria | CDR91070.1 |
| R._varieornatus_tardigradesGAU88467.1 | *Ramazzottius varieornatus* | Tardigrada | GAU88467.1 |
| R._varieornatus_tardigradesGAU93339.1 | *Ramazzottius varieornatus* | Tardigrada | GAU93339.1 |
| R._varieornatus_tardigradesGAV07616.1 | *Ramazzottius varieornatus* | Tardigrada | GAV07616.1 |
| R._bivittatum_amphibiaXP_029451037.1 | *Rhinatrema bivittatum* | Vertebrata | XP_029451037.1 |
| R._bivittatum_amphibiaXP_029465296.1 | *Rhinatrema bivittatum* | Vertebrata | XP_029465296.1 |
| R._bivittatum_amphibiaXP_029465306.1 | *Rhinatrema bivittatum* | Vertebrata | XP_029465306.1 |
| R._appendiculatus_arachnidsJAP79177.1 | *Rhipicephalus appendiculatus* | Arthropoda | JAP79177.1 |
| R._appendiculatus_arachnidsJAP86015.1 | *Rhipicephalus appendiculatus* | Arthropoda | JAP86015.1 |
| R._irregularis_FungiPKY46143.1 | *Rhizophagus irregularis* | Fungi | PKY46143.1 |
| S._trutta_bonyfishesXP_029632067.1 | *Salmo trutta* | Vertebrata | XP_029632067.1 |
| S._enterica_BacteriaCBY96094.1 | *Salmonella enterica* subsp. *enterica* serovar Weltevreden | Bacteria | CBY96094.1 |
| S._rosetta_ChoanoflagelateEGD76470.1 | *Salpingoeca rosetta* | Choanoflagellata | EGD76470.1 |
| S._rosetta_ChoanoflagelateEGD79740.1 | *Salpingoeca rosetta* | Choanoflagellata | EGD79740.1 |
| S._harrisii_mamalsXP_023363049.1 | *Sarcophilus harrisii* | Vertebrata | XP_023363049.1 |
| S._scabiei_arachnidsKPM07743.1 | *Sarcoptes scabiei* | Arthropoda | KPM07743.1 |
| S._stipitis_FungiXP_001386057.1 | *Scheffersomyces stipitis* | Fungi | XP_001386057.1 |
| S._bovis_platyhelminthesRTG84748.1 | *Schistosoma bovis* | Platyhelminthes | RTG84748.1 |
| S._haematobium_platyhelminthesKGB38128.1 | *Schistosoma haematobium* | Platyhelminthes | KGB38128.1 |
| S._japonicum_platyhelminthesCAX73753.1 | *Schistosoma japonicum* | Platyhelminthes | CAX73753.1 |
| S._japonicum_platyhelminthesCAX75389.1 | *Schistosoma japonicum* | Platyhelminthes | CAX75389.1 |
| S._japonicum_platyhelminthesTNN15762.1 | *Schistosoma japonicum* | Platyhelminthes | TNN15762.1 |
| S._culicis_EuglenozoaEPY30744.1 | *Strigomonas culicis* | Euglenozoa | EPY30744.1 |
| S._purpuratus_echinodermataXP_783413.2 | *Strongylocentrotus purpuratus* | Echinodermata | XP_783413.2 |

| | | | |
|---|---|---|---|
| S._purpuratus_echinodermataXP_011664062.1 | *Strongylocentrotus purpuratus* | Echinodermata | XP_011664062.1 |
| S._ratti_nematodaCEF67094.1 | *Strongyloides ratti* | Nematoda | CEF67094.1 |
| S._ratti_nematodaXP_024507139.1 | *Strongyloides ratti* | Nematoda | XP_024507139.1 |
| S._pistillata_cnidariaPFX23565.1 | *Stylophora pistillata* | Cnidaria | PFX23565.1 |
| S._pistillata_cnidariaXP_022795520.1 | *Stylophora pistillata* | Cnidaria | XP_022795520.1 |
| S._pistillata_cnidariaXP_022801276.1 | *Stylophora pistillata* | Cnidaria | XP_022801276.1 |
| S._suricatta_mamalsXP_029786927.1 | *Suricata suricatta* | Vertebrata | XP_029786927.1 |
| T._guttata_birdNP_001232659.1 | *Taeniopygia guttata* | Vertebrata | NP_001232659.1 |
| T._rubripes_bonyfishesXP_011620203.1 | *Takifugu rubripes* | Vertebrata | XP_011620203.1 |
| T._triunguis_ReptilaTurtlesXP_026514188.1 | *Terrapene carolina triunguis* | Vertebrata | XP_026514188.1 |
| T._thermophila_CiliatesEAR98203.3 | *Tetrahymena thermophila* | Ciliophora | EAR98203.3 |
| T._thermophila_CiliatesXP_001031817.3 | *Tetrahymena thermophila* | Ciliophora | XP_001031817.3 |
| T._urticae_arachnidsXP_015787653.1 | *Tetranychus urticae* | Arthropoda | XP_015787653.1 |
| T._lacteum_AmoebozoaKYR00648.1 | *Tieghemostelium lacteum* | Amoebozoa | KYR00648.1 |
| T._canis_nematodaKHN84477.1 | *Toxocara canis* | Nematoda | KHN84477.1 |
| T._nativa_nematodaOUC47719.1 | *Trichinella nativa* | Nematoda | OUC47719.1 |
| V._destructor_arachnidsXP_022654745.1 | *Varroa destructor* | Arthropoda | XP_022654745.1 |
| V._ursinus_mamalsXP_027701613.1 | *Vombatus ursinus* | Vertebrata | XP_027701613.1 |
| X._tropicalis_amphibiaNP_989352.1 | *Xenopus tropicalis* | Vertebrata | NP_989352.1 |
| X._tropicalis_amphibiaNP_001119993.1 | *Xenopus tropicalis* | Vertebrata | NP_001119993.1 |
| Z._californianus_mamalsXP_027425207.1 | *Zalophus californianus* | Vertebrata | XP_027425207.1 |
| Z._tritici_FungiEGP83856.1 | *Zymoseptoria tritici* | Fungi | EGP83856.1 |

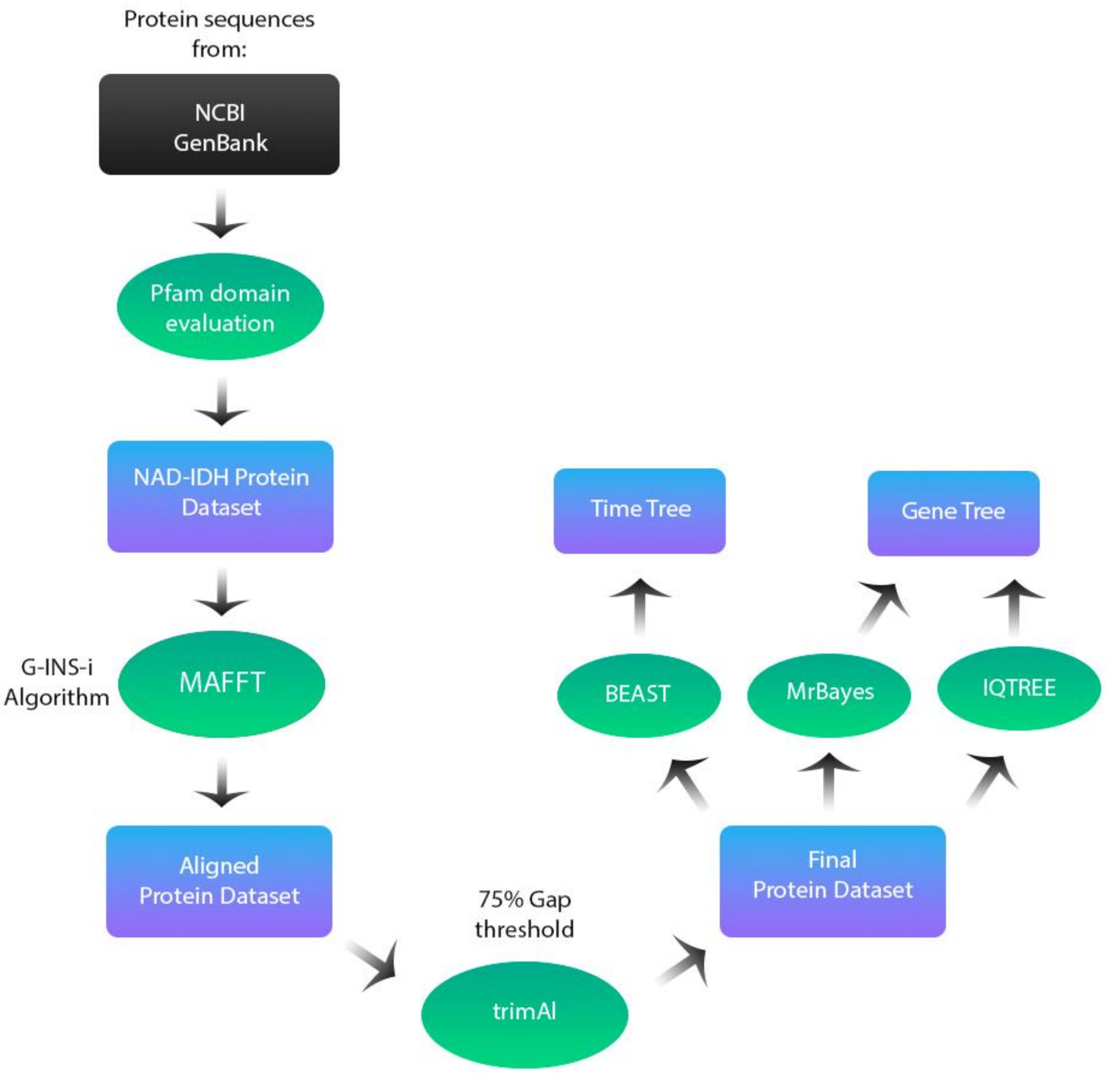


**Supplementary Figure S2** – Bioinformatic pipeline. Ovals represent software or scripts, and rounded rectangles represent input/output files. The dataset was formed using 195 previously published NAD-IDH sequences distributed as: 193 eukaryotic sequences and two bacterial.

**Supplementary Figure S3** – Credidibility intervals and node ages estimated based on a mixture model in PhyloBayes and ones inferred by BEAST.

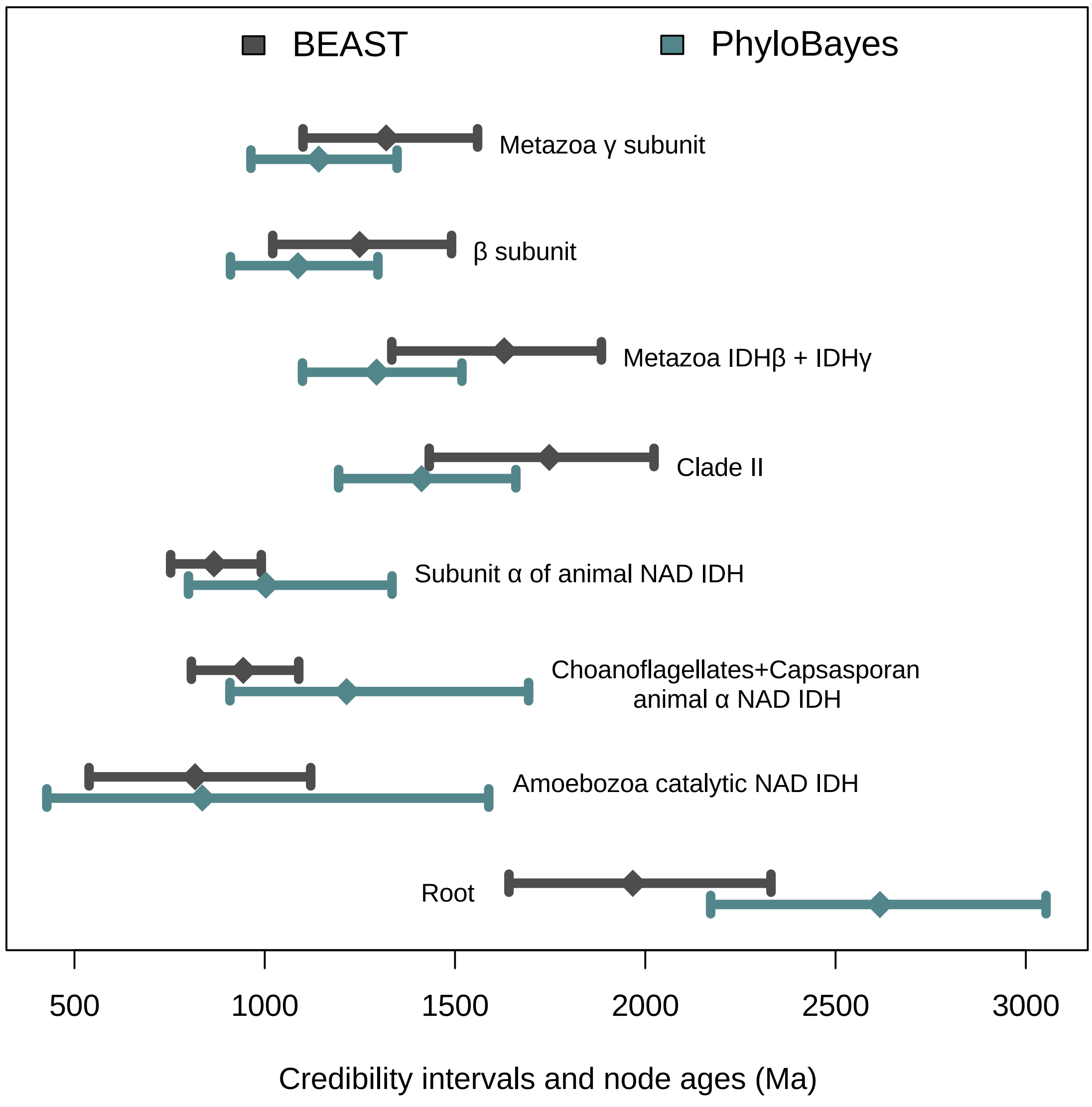

Supplement: Supplementary file 1 — Supplementary Informations [file 41598_2021_95094_MOESM1_ESM.pdf]
